# Protective Role of Nuclear Factor E2-Related Factor 2 against Acute Oxidative Stress-Induced Pancreatic ***β***-Cell Damage

**DOI:** 10.1155/2015/639191

**Published:** 2015-04-09

**Authors:** Jingqi Fu, Hongzhi Zheng, Huihui Wang, Bei Yang, Rui Zhao, Chunwei Lu, Zhiyuan Liu, Yongyong Hou, Yuanyuan Xu, Qiang Zhang, Weidong Qu, Jingbo Pi

**Affiliations:** ^1^Program of Environmental Toxicology, School of Public Health, China Medical University, No. 77 Puhe Road, Shenyang North New Area, Shenyang, Liaoning 110122, China; ^2^The First Affiliated Hospital, China Medical University, 155 Nanjingbei Street, Heping District, Shenyang, Liaoning 110001, China; ^3^Department of Histology and Embryology, School of Basic Medical Sciences, China Medical University, No. 77 Puhe Road, Shenyang North New Area, Shenyang, Liaoning 110122, China; ^4^School of Forensic Medicine, China Medical University, No. 77 Puhe Road, Shenyang North New Area, Shenyang, Liaoning 110122, China; ^5^Institute for Chemical Safety Sciences, The Hamner Institutes for Health Sciences, 6 Davis Drive, Research Triangle Park, NC 27709, USA; ^6^Key Laboratory of Public Health Safety of Ministry of Education, School of Public Health, Fudan University, P.O. Box 249, 138 Yi Xue Yuan Road, Shanghai 200032, China

## Abstract

Oxidative stress is implicated in the pathogenesis of pancreatic *β*-cell dysfunction that occurs in both type 1 and type 2 diabetes. Nuclear factor E2-related factor 2 (NRF2) is a master regulator in the cellular adaptive response to oxidative stress. The present study found that MIN6 *β*-cells with stable knockdown of *Nrf2* (*Nrf2*-KD) and islets isolated from *Nrf2*-knockout mice expressed substantially reduced levels of antioxidant enzymes in response to a variety of stressors. In scramble MIN6 cells or wild-type islets, acute exposure to oxidative stressors, including hydrogen peroxide (H_2_O_2_) and S-nitroso-N-acetylpenicillamine, resulted in cell damage as determined by decrease in cell viability, reduced ATP content, morphology changes of islets, and/or alterations of apoptotic biomarkers in a concentration- and/or time-dependent manner. In contrast, silencing of *Nrf2* sensitized MIN6 cells or islets to the damage. In addition, pretreatment of MIN6 *β*-cells with NRF2 activators, including CDDO-Im, dimethyl fumarate (DMF), and *tert*-butylhydroquinone (tBHQ), protected the cells from high levels of H_2_O_2_-induced cell damage. Given that reactive oxygen species (ROS) are involved in regulating glucose-stimulated insulin secretion (GSIS) and persistent activation of NRF2 blunts glucose-triggered ROS signaling and GSIS, the present study highlights the distinct roles that NRF2 may play in pancreatic *β*-cell dysfunction that occurs in different stages of diabetes.

## 1. Introduction

Pancreatic *β*-cell damage is the fundamental pathogenesis of type 1 diabetes, which is mediated by an autoimmune and inflammatory process [1]. Disruption of pancreatic *β*-cells leading to islet dysfunction and reduced *β*-cell mass is also implicated in type 2 diabetes [2]. Oxidative stress, which is characterized by increased production of reactive oxygen species (ROS) and/or reactive nitrogen species (RNS), is involved in the destruction of *β*-cells in different stages of both type 1 and type 2 diabetes. Although ROS, in particular hydrogen peroxide (H_2_O_2_), may function as intracellular secondary messenger mediating glucose-stimulated insulin secretion (GSIS) in pancreatic *β*-cells [3–5], excessive and persistent production of ROS results in oxidative damage and disrupts the function of proteins, nucleic acids, and fatty acids [6, 7].

In type 1 diabetes, pancreatic *β*-cell failure is mediated by inflammatory cytokines, including interleukin-1*β* (IL-1*β*), tumor necrosis factor-*α* (TNF-*α*), and interferon-*γ* (IFN-*γ*), which may stimulate production of RNS, especially nitric oxide (NO) [8]. Similar to H_2_O_2_, NO also functions as an important messenger molecule that mediates a variety of physiological processes, including vasodilation [9], neurotransmission [10], and immunity response [11]. However, persistent elevation of endogenous or exogenous RNS may also induce cytotoxic effects in various cell types. High levels of NO disrupt energy metabolism, induce DNA damage, activate poly(ADP-ribose) polymerase (PARP), and/or dysregulate cytosolic calcium leading to cell necrosis and apoptosis [12, 13].

Although ROS and RNS participate in various cellular response pathways, they both induce DNA damage and reduce cell viability at high levels [14]. Of interest, cells have acquired complicated mechanisms to localize endogenous ROS and/or RNS distribution and defend against exogenous ROS and/or RNS toxicity. Among them, transcriptional signaling through the antioxidant response element (ARE), orchestrated by the nuclear factor E2-related factor 2 (NRF2), is a major cellular defense mechanism against oxidative or electrophilic stress.* Nrf2*-deficient animals and cells have been shown to display reduced antioxidant response and become intolerant to oxidative or electrophilic stress-induced damage [15–19]. Compared with other cell types and tissues, pancreatic *β*-cells express low levels of many antioxidant enzymes and thus are hypothesized to be susceptible to oxidative damage induced by ROS and/or RNS [20]. However, the role of NRF2-mediated antioxidant response in acute ROS or RNS-induced pancreatic *β*-cell damage has not been thoroughly investigated.

In the current study, we hypothesized that NRF2 plays a protective role against acute oxidative stress-induced pancreatic *β*-cell damage, a process that may occur in type 1 and type 2 diabetes. To test the hypothesis, we determined the susceptibility of* Nrf2*-knockout (*Nrf2*−/−) islets and* Nrf2*-knockdown (*Nrf2*-KD) MIN6 cells to high levels of oxidative stressors-induced cell damage. In addition, we investigated the protective effects of preactivation of NRF2 on acute oxidative stress-induced cell damage.

## 2. Materials and Methods

### 2.1. Cell Culture and Reagents

MIN6 cells at passage 30 were kindly gifted by Dr. Marcia Haigis (Harvard University, USA) and maintained in Dulbecco's modified Eagle's medium (DMEM) containing 25 mmol/l glucose, supplemented with 15% fetal bovine serum (FBS), 100 units/mL penicillin, 100 *μ*g/mL streptomycin, 2 mmol/l L-glutamine, and 5 *μ*L/l *β*-mercaptoethanol in humidified 5% CO_2_, 95% air at 37°C as previously described [21]. The cells at passages 42–48 were used in the current study. There was no significant difference among the passages in their glucose responsiveness and cytotoxic susceptibility to a variety of stressors. H_2_O_2_ solution, glucose oxidase, S-nitroso-N-acetylpenicillamine (SNAP), sodium arsenite,* tert*-butylhydroquinone (tBHQ), *β*-mercaptoethanol, hexadimethrine bromide, dimethyl fumarate (DMF), and 1-[2-cyano-3,12-dioxooleana-1,9(11)-dien-28-oyl]imidazole (CDDO-Im) were purchased from Sigma (Saint Louis, MO).

### 2.2. Establishment of Stable Cell Lines and Measurement of Luciferase Activity

MIN6 cells were transduced with* Lentivirus* containing shRNA against* Nrf2* (SHVRSNM_010902, Sigma), scrambled nontarget negative control (SHC002V, Sigma), or ARE-luciferase reporter (SABiosciences, Frederick, MD) as previously described [17]. The luciferase activity of ARE-luciferase reporter cells was determined by Luciferase Reporter Assay System (Promega, Madison, WI) and normalized to cell viability with the same treatment as detailed in our previous studies [22].

### 2.3. Animals and Islet Isolation

*Nrf2*−/− mice developed as described previously [23] were kindly provided by Dr. Masayuki Yamamoto (Tohoku University, Japan). Wild-type (*Nrf2*+/+) and* Nrf2*−/− mice were maintained on normal chow diet. All animal experiments were approved by the Institutional Animal Care and Use Committee of China Medical University. Pancreatic islets were isolated from 8–12-week-old* Nrf2*+/+ and* Nrf2*−/− mice by* in situ* collagenase P (Roche, Switzerland) perfusion of pancreas under a dissecting microscope, as described previously [4]. Isolated islets were cultured in RPMI 1640 supplemented with 10 mmol/l glucose, 10% FBS, 25 mmol/l HEPES, 2 mmol/l L-glutamine, 100 U/mL penicillin, and 100 *μ*g/mL streptomycin. All the isolated islets were cultured for 48 hrs before* in vitro* experiments.

### 2.4. Measurement of Intracellular Glutathione (GSH)

MIN6 cells were sonicated in cold PBS immediately after collection. The whole cell extracts were obtained by centrifugation at 12,000 ×g for 5 min, and protein concentration was determined by a BCA assay kit (Pierce Biotechnology, Rockford, IL). Following an immediate deproteinization with metaphosphoric acid (final concentration at 5%), levels of total GSH were measured using a BIOXYTECH GSH/GSSG-412 kit (OxisResearch, Portland, OR) according to the manufacturer's protocol. Of note, the levels of oxidized GSH (GSSG) in MIN6 cells were too low to be measured.

### 2.5. Determination of Intracellular Peroxide

Levels of intracellular peroxide were measured by flow cytometry (Becton Dickinson FACSort, Becton Dickinson, San Jose, CA) using a fluorescent probe 5-(and-6)-chloromethyl-2′,7′-dichlorodihydrofluorescein diacetate, acetyl ester (CM-H_2_DCFDA, Molecular Probes, Eugene, OR) as described previously [4]. The loading concentration of CM-H_2_DCFDA was 2 *μ*M and the preloading times were 30 min. In the measurements, dead cells and clumps were eliminated based upon Forward Scatter versus Side Scatter measurements, and untreated cells provided a source of comparison.

### 2.6. Quantitative Real-Time RT-PCR

Total RNA was extracted from MIN6 cells and isolated islets with TRIzol reagent (Life Technologies, Carlsbad, CA) and purified by RNeasy Mini kit and RNase-Free DNase Set (Qiagen, Valencia, CA). cDNA was prepared by MuLV reverse transcriptase using Oligo (dT) primers (Applied Biosystems, Foster City, CA). Relative mRNA abundance was determined by quantitative real-time PCR with SYBR green detection using ABI PRISM 7900HT Fast Real-Time PCR System as described previously [24]. The primers were designed using the Primer Express (Applied Biosystems) and synthesized by MWG-BIOTECH Inc. (High Point, NC). The sequences are shown in Table 1.

### 2.7. Analysis of Proteins

Isolation of cell fraction and immunoblotting were performed as detailed previously [24, 25]. Briefly, whole cell extracts were prepared by sonication with cell lysis buffer (Cell Signaling, Technology, Inc., Beverly, MA) with Protease Inhibitor Cocktail (Sigma) and Phosphatase Inhibitor Cocktail I (Sigma). Nuclear fractions were separated by the TransFactor Extraction Kit (BD Biosciences Clontech, Palo Alto, CA) according to the manufacturer's protocols. Protein concentrations were determined by BCA assay kit (Pierce), and all the protein fractions were stored at −80°C until use. Protein extracts were boiled and resolved on Novex Tris-Glycine Gel (Life Technologies), transferred onto nitrocellulose membranes (Bio-Rad) for immunoblotting, and visualized by ECF substrate (GE Healthcare). Blot imaging and quantification were performed using a Typhoon scanner and the Bio-Rad TDS Quantity One software. Antibodies for NRF2 (sc-13032; 1 : 500) were purchased from Santa Cruz Biotechnology, Inc. (Santa Cruz, CA). Antibodies for cleaved Caspase-3 (number 9661, 1 : 1000), cleaved PARP (number 9542, 1 : 1000), and Caspase-3 (number 9662, 1 : 1000) were from Cell Signaling Technology, Inc. (Danvers, MA). Antibodies for Lamin A (L1293; 1 : 2500) and *β*-Actin (A1978; 1 : 2000) were purchased from Sigma.

### 2.8. Cell Viability and Cytotoxicity Assay

Cell viability was determined as described previously [26]. Briefly, 10,000 cells per well were plated into a 96-well plate. Following a 24 hr culture, medium was replaced with fresh medium containing various stressors at the appropriate concentrations. Cells were treated for indicated time with stressors and followed by an immediate measurement for cell viability by using the nonradioactive cell proliferation assay kit (Promega, Madison, WI). Intracellular ATP levels were measured using an ATP Bioluminescent Somatic Cell Assay Kit (Sigma).

### 2.9. Statistical Analyses

All statistical analyses were performed using Graphpad Prism 4 (GraphPad Software, San Diego, CA). Statistical significance was defined as *P* < 0.05. Data are expressed as mean ± SD. For comparisons between and among groups, Student's *t*-test and one-way ANOVA with Bonferroni post hoc testing were performed, respectively.

## 3. Results

### 3.1. Stable Knockdown of* Nrf2* in MIN6 Cells Results in Attenuated Antioxidant Response

**Figure 1:**
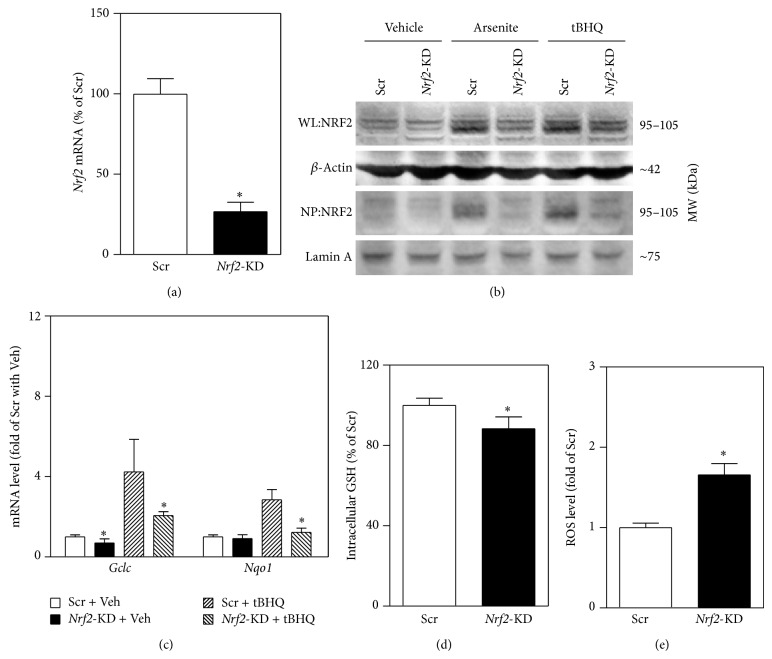
Stable knockdown of* Nrf2* results in reduced expression of ARE-dependent genes, decreased intracellular GSH, and elevated intracellular ROS levels in MIN6 cells. (a) mRNA expression of* Nrf2* in cells transduced with lentiviral shRNA targeted against mouse* Nrf2 *(*Nrf2*-KD) or scrambled nontarget negative control (Scr). (b) Reduced protein expression of NRF2 in* Nrf2*-KD cells under basal and arsenite (5 *μ*M),* tert*-butylhydroquinone (tBHQ, 50 *μ*M) treated conditions. Cells were exposed to the chemicals for 6 hrs. Whole cell lysates (WL) and nuclear protein (NP) were used for immunoblotting, and *β*-Actin and Lamin A were used as loading controls, respectively. (c) Expression of ARE-dependent genes under vehicle (Veh) and tBHQ treated conditions. Cells were treated with the tBHQ for 6 hrs. The expression of *Gclc* and* Nqo1* was measured by real-time RT-PCR. (d) Intracellular GSH levels. (e) Intracellular ROS levels. Values in (a), (c), (d), and (e) are means ± SD. *n* = 3–6. ^∗^ *P* < 0.05 versus Scr with the same treatment.

To investigate the role of NRF2 in acute oxidative stress-induced *β*-cell damage, a line of MIN6 cells with stable silencing of* Nrf2* was developed. As shown in Figure 1(a), lentiviral shRNA-mediated stable knockdown of* Nrf2* in MIN6 cells resulted in 70% reduction in mRNA expression of* Nrf2* compared to the control cells that were expressed scrambled nontarget negative control shRNA (scramble). In agreement with the reduction of* Nrf2* mRNA,* Nrf2*-KD cells displayed substantially reduced protein expression of NRF2 under vehicle and arsenite- or tBHQ-challenged conditions (Figure 1(b)). In scramble cells, the protein levels of NRF2 in nuclear fractions were dramatically increased by arsenite or tBHQ treatments, whereas the inductions were almost totally diminished in* Nrf2*-KD cells (Figure 1(b)). In addition,* Nrf2*-KD cells showed reduced expression of two major ARE-dependent genes glutamate-cysteine ligase catalytic subunit (*Gclc*) and NAD(P)H quinone oxidoreductase 1 (*Nqo1*) under basal or tBHQ-challenged conditions (Figure 1(c)). Furthermore, silencing of* Nrf2* resulted in significantly decreased intracellular levels of GSH (Figure 1(d)) and elevation of intracellular ROS (Figure 1(e)).

### 3.2. *Nrf2*-Deficient *β*-Cells Are Vulnerable to Acute Oxidative Stress-Induced Cell Damage

**Figure 2:**
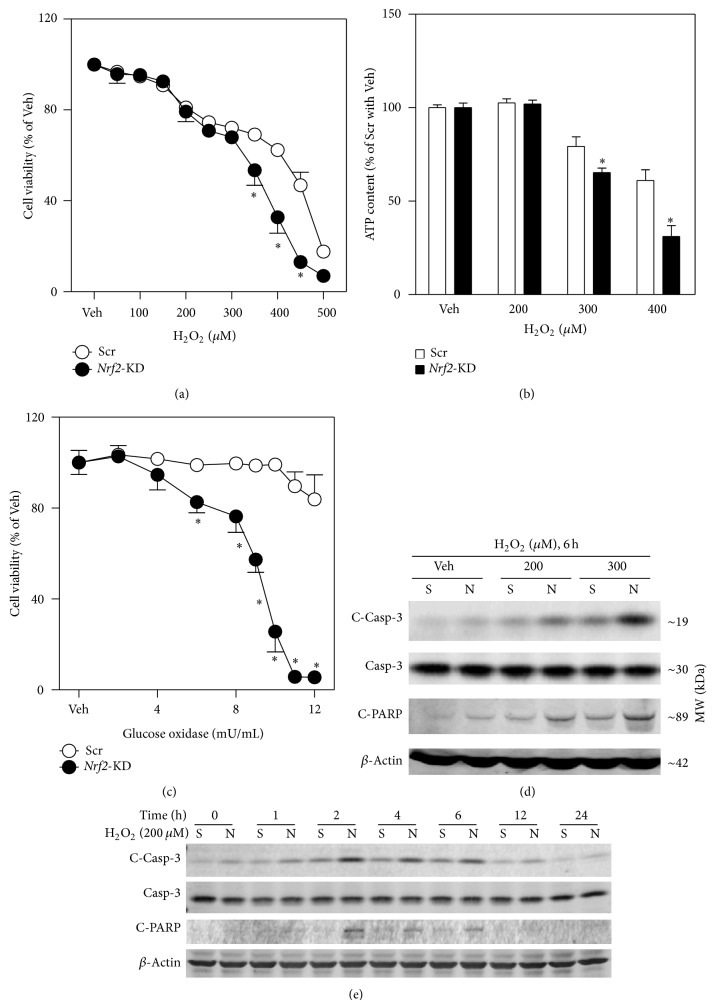
*Nrf2* silencing sensitizes MIN6 cells to oxidative stressor-induced cell damage. ((a) and (c)) Concentration-dependent decrease of cell viability in* Nrf2*-KD and Scr cells caused by H_2_O_2_ (a) and glucose oxidase (c) exposure. Cell viability was assessed by MTT assay following a 24 hr treatment. (b) ATP content in Scr and* Nrf2*-KD cells following a 24 hr H_2_O_2_ treatment. ((d) and (e)) Immunoblotting of cleaved Caspase-3 (C-Casp-3), Caspase-3 (Casp-3), and cleaved PARP (C-PARP) in MIN6 cells exposed to H_2_O_2_ for indicated time and concentrations. *β*-Actin was used as a loading control. S, Scr; N,* Nrf2*-KD. Values in (a), (b), and (c) are means ± SD. *n* = 3–6. ^∗^ *P* < 0.05 versus Scr with the same treatment.

To test the hypothesis that NRF2 plays a protective role against the cytotoxicity of acute oxidative stress in pancreatic *β*-cells, we investigated the susceptibility of* Nrf2*-KD MIN6 cells and* Nrf2*−/− mouse islets to H_2_O_2_-induced cell damage. As shown in Figure 2,* Nrf2*-KD cells were more vulnerable to H_2_O_2_ solution-induced reduction in cell viability (Figure 2(a)) and ATP content (Figure 2(b)). In response to glucose oxidase, which is a mild and long-lasting H_2_O_2_-generating system that catalyzes the oxidation of glucose to produce H_2_O_2_,* Nrf2*-KD MIN6 cells also showed an elevated susceptibility to its cytotoxicity (Figure 2(c)). Of note, application of glucose oxidase may reduce the concentrations of glucose in media, which may directly affect cell viability. Thus, the glucose oxidase-induced cytotoxicity in Scr and* Nrf2*-KD cells could be a mixed effect of glucose removal and H_2_O_2_ exposure. Consistent with the assays for cytotoxicity, acute H_2_O_2_ exposure resulted in dramatically increased levels of multiple apoptotic markers, including cleaved Caspase-3 (C-Casp-3) and cleaved PARP (C-PARP), in Scr and* Nrf2*-KD cells in a concentration- and time-dependent fashion (Figures 2(d) and 2(e)). Importantly, the H_2_O_2_ exposure-induced increase of these apoptotic markers in* Nrf2*-KD was substantially higher than that in Scr cells (Figures 2(d) and 2(e)), indicating that deficiency of* Nrf2 *makes *β*-cells susceptible to H_2_O_2_ exposure-induced apoptosis.

**Figure 3:**
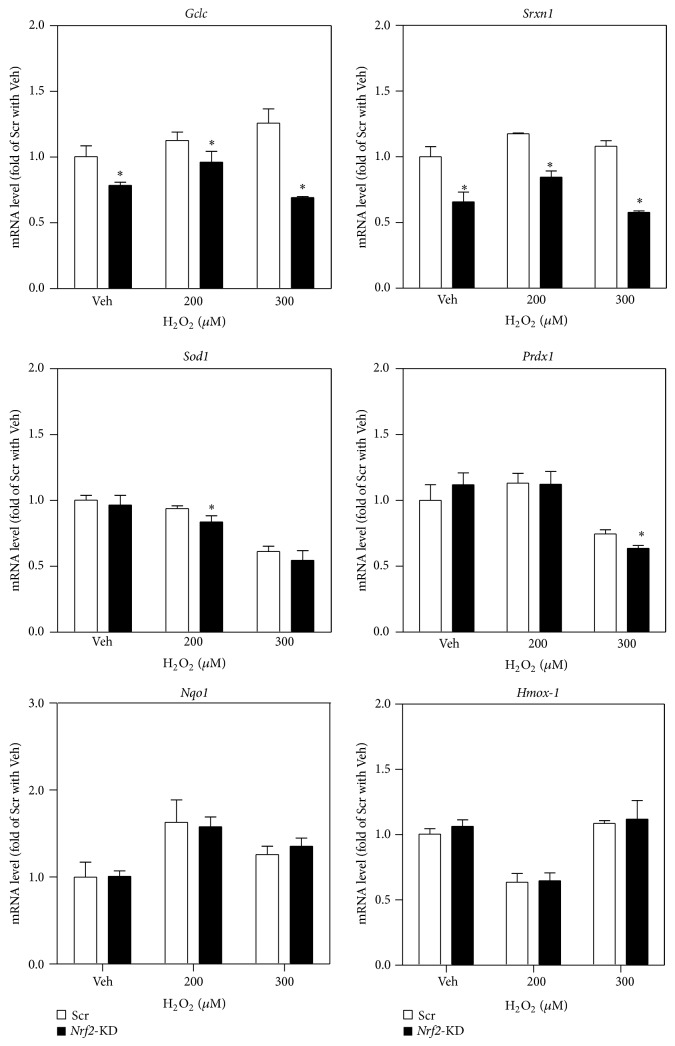
Reduced expression of antioxidant genes in* Nrf2*-KD cells under basal and H_2_O_2_-treated conditions. Cells were treated with H_2_O_2_ for 6 hrs. Values are means ± SD. *n* = 3–6. ^∗^ *P* < 0.05 versus Scr with the same treatment.

To ascertain the involvement of NRF2-mediated antioxidant response in protection against acute H_2_O_2_-induced cell damage, the expression of multiple antioxidant genes, including* Gclc,* sulfiredoxin (*Srxn1*), superoxide dismutase-1 (*Sod1)*, peroxiredoxin-1 (*Prdx1)*,* Nqo1,* and heme oxygenase 1 (*Hmox-1*), was determined in* Nrf2*-KD and scramble MIN6 cells under basal and H_2_O_2_-challenged conditions. As shown in Figure 3, mRNA levels of* Gclc, Srxn1, Sod1, and Prdx1 *were significantly attenuated by* Nrf2* silencing under basal or H_2_O_2_-challenged conditions, suggesting that the NRF2-ARE system is important in determining cell fate in response to acute oxidative stress. However, there was no significant induction of the ARE-dependent genes in response to H_2_O_2_ challenge in Scr cells, suggesting that H_2_O_2_ is not a strong NRF2 activator at low concentrations (<0.3 mM) in MIN6 cells. In addition, the mRNA expression of* Nqo1* and* Hmox-1* showed no significant difference between Scr and* Nrf2*-KD cells under H_2_O_2_ treatment, which is distinct from the patterns induced by other stressors, including tBHQ (Figure 1(c)) and arsenite [17].

**Figure 4:**
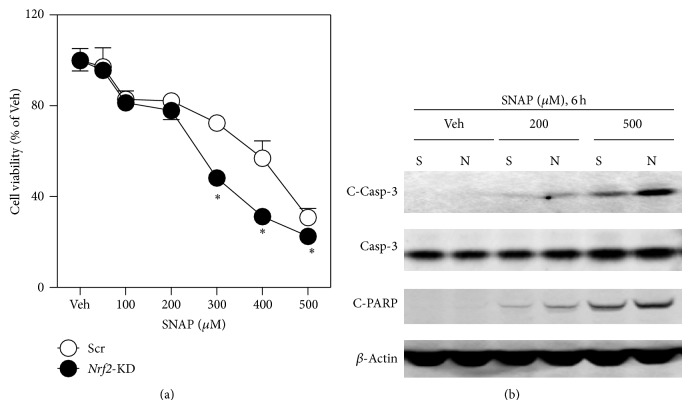
*Nrf2*-KD MIN6 cells are vulnerable to SNAP-induced cytotoxicity. (a) Following a 24 hr exposure to SNAP cell viability was assessed by MTT assay. (b) Immunoblotting of cleaved Caspase-3 (C-Casp-3), Caspase-3 (Casp-3), and cleaved PARP (C-PARP) in MIN6 cells exposed to SNAP for indicated time and concentrations. *β*-Actin was used as a loading control.

To extend the findings above, the susceptibility of scramble and* Nrf2*-KD MIN6 cells to a NO-releasing compound SNAP-induced cytotoxicity was determined. Compared to Scr cells,* Nrf2*-KD MIN6 cells were more vulnerable to SNAP-induced reduction in cell viability (Figure 4(a)) and apoptosis (Figure 4(b)).

**Figure 5:**
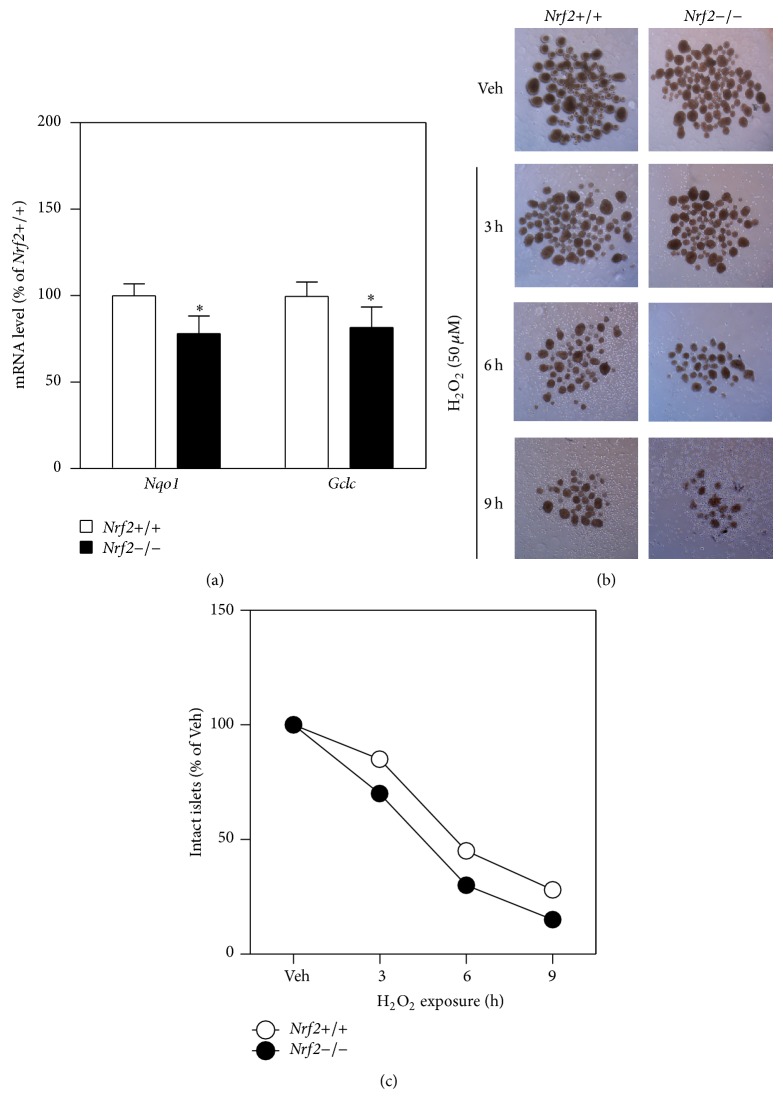
Deficiency of* Nrf2* results in reduced expression of antioxidant genes and sensitization to H_2_O_2_-induced damage in isolated mouse islets. (a)* Nrf2*−/− islets exhibit reduced expression of antioxidant genes. Total RNA was extracted from isolated islets that were cultured for 48 hrs. *n* = 3. ^∗^ *P* < 0.05 versus* Nrf2*+/+ islets. (b) Representative images of cultured islets that have been exposed to 50 *μ*M H_2_O_2_ for indicated time. The islets were examined by an Olympus SZX7 Zoom Stereomicroscope (4x). (c) Count of intact islets left following H_2_O_2_ exposure as shown in (b).

To validate the key findings observed in MIN6 cells, H_2_O_2_-induced cell damage was determined in cultured islets isolated from wild-type and* Nrf2*−/− mice. As shown in Figure 5,* Nrf2*−/− islets showed reduced expression of* Nqo1* and* Gclc* (Figure 5(a)), confirming that the ARE-dependent transcription was attenuated in the tissues. Consistent with the conclusions obtained in MIN6 cells,* Nrf2*−/− islets exhibited more serious damage than* Nrf2*+/+ islets as determined by morphology changes in response to H_2_O_2_ exposure (Figures 5(b) and 5(c)).

### 3.3. Preactivation of NRF2 Protects MIN6 Cells against H_2_O_2_-Induced Cytotoxicity and Apoptosis

**Figure 6:**
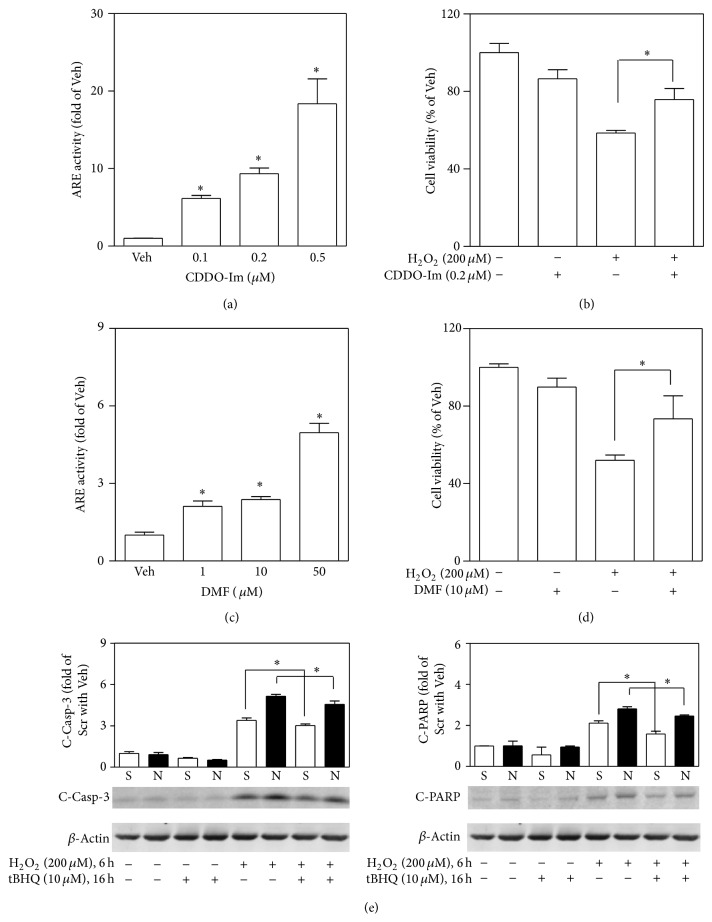
Preactivation of NRF2 protects MIN6 cells from H_2_O_2_-induced cytotoxicity. ((a) and (c)) ARE-luciferase activity measured in MIN6 cells. The cells were challenged with CDDO-Im (a) or DMF (b) for 4 hrs. ((b) and (d)) Effects of CDDO-Im and DMF pretreatment on H_2_O_2_-induced cytotoxicity. Following a 4 hr pretreatment with CDDO-Im (b) or DMF (d) cells were exposed to H_2_O_2_ for 12 hrs. Cell viability was assessed by MTT assay. (e) Cells were pretreated with 10 *μ*M tBHQ for 16 hrs followed by a 6 hr H_2_O_2_ exposure. Apoptosis markers cleaved Caspase-3 (C-Casp-3) and cleaved PARP (C-PARP) were determined using immunoblotting and quantified by ImageJ 1.44. *n* = 3. ^∗^ *P* < 0.05 versus nonpretreated cells with the same treatment.

To further determine the protective role of NRF2-mediated antioxidant response in acute oxidative stress-induced *β*-cell damage, the effects of preactivation of NRF2 by multiple NRF2 activators on H_2_O_2_-induced cytotoxicity and apoptosis were determined in MIN6 cells. As shown in Figure 6, a 4 hr pretreatment with either CDDO-Im (Figure 6(a)) or DMF (Figure 6(c)) significantly enhanced ARE-luciferase activity in a concentration-dependent manner, indicating both compounds are potent activators of NRF2-mediated antioxidant response in the cells. Pretreatment of MIN6 cells individually with these two activators significantly attenuated H_2_O_2_-induced cytotoxicity (Figures 6(b) and 6(d)). In addition, pretreatment with another NRF2 activator, tBHQ (10 *μ*M), also substantially protected MIN6 cells from the H_2_O_2_-induced apoptosis as measured by elevation of C-Casp-3 and C-PARP (Figure 6(e)). Thus, preactivation of NRF2 by either CDDO-Im, DMF, or tBHQ may partially protect MIN6 cells against H_2_O_2_-induced acute cell damage.

## 4. Discussion

The impairment of pancreatic *β*-cell function is critical in the pathophysiology of both type 1 and type 2 diabetes. Amidst the various mechanisms proposed for *β*-cell dysfunction and their roles in the progression of *β*-cell damage, oxidative stress has been proposed as a common denominator [27]. The present study found that acute exposure of MIN6 *β*-cells or isolated mouse islets to the most common oxidative stressors, H_2_O_2_ or NO, resulted in cell damage in a concentration- and time-dependent manner.* Nrf2*-KD MIN6 *β*-cells and* Nrf2*−/− islets were vulnerable to H_2_O_2_ or SNAP-induced cytotoxicity and apoptosis. In addition, pretreatment of MIN6 *β*-cells with a variety of NRF2 activators protected the cells from high levels of H_2_O_2_-induced cell damage. These findings clearly demonstrate that NRF2-mediated antioxidant response protects *β*-cells from acute oxidative stress-induced cell damage.

Oxidative stress in pancreatic *β*-cells is generally induced by glucotoxicity, lipotoxicity, and/or inflammation in different stages of diabetes [27]. Although ROS and RNS are critical signaling molecules mediating a variety of signaling cascades [14], they both lead to cell damage at high levels. Partially due to low basal expression of many antioxidant enzymes, *β*-cells are relatively vulnerable to oxidative damage induced by excessive ROS and/or RNS [20]. In the present study, we found that H_2_O_2_ may induce cell damage in MIN6 cells and cultured islets at a concentration as low as 50 *μ*M, which is dramatically lower than the concentrations we used in human keratinocytes to activate NRF2 [25]. Under oxidative stress, most of cells may upregulate their intracellular antioxidant capacity by transcriptional induction of many antioxidant and phase II detoxification enzymes via NRF2-mediated antioxidant response and thus protect the cells from oxidative damage [15, 18, 19, 28–30]. Our previous studies have elucidated that NRF2 also plays a critical role in pancreatic *β*-cell defense against oxidative/electrophilic stress [17]. Abolishment of the NRF2-mediated antioxidant response by targeted disruption of the* Nrf2* gene in MIN6 cells and mouse islets increased their susceptibility to environmental oxidative stressor arsenic-induced cytotoxicity and/or apoptosis [17]. Preactivation of NRF2 with tBHQ significantly protects MIN6 cells from arsenic-induced acute cytotoxicity in* Nrf2*-dependent manner [17]. Recently, Yagishita et al. presented detailed* in vivo* evidence from four genetically engineered mouse models to demonstrate that NRF2 induction prevents oxidative and nitrosative stress-induced oxidative damage in pancreatic *β*-cells [31]. In the current study we used the most common oxidative stressors and two genetically engineered cell models to confirm that NRF2-mediated antioxidant response protects pancreatic *β*-cell against oxidative stress-induced cell damage* in vitro* and* ex vivo*. These new findings provided additional evidence to support that NRF2-mediated antioxidant response protects pancreatic *β*-cell against acute ROS or RNS-induced cell damage.

Given that NRF2-mediated antioxidant response plays critical role in cell defense, NRF2 has been considered as a valuable therapeutic target. CDDO-Im is one of the most potent synthetic triterpenoids shown to activate NRF2 and induce phase II detoxifying and antioxidant enzymes. It was firstly investigated in protecting against aflatoxin-induced tumorigenesis in liver [32, 33]. Recently, it has been used for the treatment of chronic kidney disease, cancer, and other diseases [34–36]. DMF is a newly found NRF2 activator and has been used for the treatment of psoriasis [37] and more recently for multiple sclerosis [38]. CDDO-Im and DMF have been reported to protect endothelial cells and prevent vascular injury via NRF2 activation [39, 40]. In the present study, we found that CDDO-Im and DMF pretreatments may dramatically enhance the activity of ARE-dependent transcription in pancreatic *β*-cells and protect the cells against acute oxidative stress-induced cell damage. Together with the protective effect by another well-known NRF2 activator, tBHQ, we conclude that preactivation of NRF2 may protect pancreatic *β*-cells from acute oxidative stress-induced cell damage.

Although ROS and RNS have been demonstrated to be destructive factors at high levels, they also function as physiological signaling molecules mediating a variety of physiological processes, including GSIS in *β*-cells [3, 4]. In the case where ROS serve as cell signaling molecules, persistent elevation of endogenous antioxidants could diminish such a signal. Thus, persistent activation of NRF2-mediated antioxidant response has the potential to cause an undesirable effect. Our previous study showed that persistent NRF2 activation by prolonged arsenic exposure blunts glucose-stimulated ROS signaling to mediate GSIS in pancreatic *β*-cells [22]. Therefore, we have proposed that NRF2-mediated antioxidant response plays paradoxical roles in *β*-cell function. On the one hand, this pathway protects cells from oxidative damage and possible cell death, thus minimizing oxidative damage-related impairment in *β*-cell dysfunction; on the other hand, the induction of endogenous antioxidants in the presence of oxidative stress may blunt ROS signal, resulting in reduced GSIS [41, 42]. Considering that glucose metabolism-derived ROS are involved in regulating GSIS in *β*-cells and persistent activation of NRF2 blunts glucose-triggered ROS signaling and GSIS, we conclude that NRF2 may play distinct roles in pancreatic *β*-cell dysfunction that occurs in different stages of diabetes.

## 5. Conclusions

The present study demonstrated that NRF2-mediated antioxidant response protects pancreatic *β*-cells from oxidative stress-induced cell damage. In light of the inhibitory effect of NRF2-mediated antioxidant response on ROS signaling in *β*-cell GSIS, the present study highlights distinct roles that NRF2 may play in pancreatic *β*-cell dysfunction that occurs in different stages of diabetes. Thus, more detailed investigations focusing on the exact mode of ROS and various endogenous antioxidants in regulating GSIS and *β*-cell survival during different stages of diabetes are needed.

## Figures and Tables

**Table 1:** Primer sequences for real-time RT-PCR.

Gene	Sense	Antisense
*18s *	CGAACGTCTGCCCTATCAACTT	CCGGAATCGAACCCTGATT
*Gclc *	TGGCCACTATCTGCCCAATT	GTCTGACACGTAGCCTCGGTAA
*Hmox-1 *	CCTCACTGGCAGGAAATCATC	CCTCGTGGAGACGCTTTACATA
*Nqo1 *	TATCCTTCCGAGTCATCTCTAGCA	TCTGCAGCTTCCAGCTTCTTG
*Nrf2 *	CGAGATATACGCAGGAGAGGTAAGA	GCTCGACAATGTTCTCCAGCTT
*Prdx1 *	TCCCAAGCGCACCATTG	AGGCCCCTGAAAGAGATACCTT
*Sod1 *	GTGATTGGGATTGCGCAGTA	TGGTTTGAGGGTAGCAGATGAGT
*Srxn1 *	CCGTTCTTCAGTCTATGAAAAGATAACA	CCGAGTCCATCTTTCTTACTTCCT

## Abbreviations

ARE: Antioxidant response element
CDDO-Im: 1-[2-Cyano-3,12-dioxooleana-1,9(11)-dien-28-oyl]imidazole
C-Casp-3: Cleaved Caspase-3
CM-H_2_DCFDA: 5-(and-6)-Chloromethyl-2′,7′-dichlorodihydrofluorescein diacetate, acetyl ester
C-PARP: Cleaved PARP
DMEM: Dulbecco's modified Eagle's medium
DMF: Dimethyl fumarate
FBS: Fetal bovine serum
*Gclc*: Glutamate-cysteine ligase catalytic subunit
GSH: Glutathione
GSIS: Glucose-stimulated insulin secretion
*Hmox-1*: Heme oxygenase 1
H_2_O_2_: Hydrogen peroxide
NO: Nitric oxide
*Nqo1*: NAD(P)H quinone oxidoreductase 1
*Nrf2*: Nuclear factor E2-related factor 2
PARP: Poly(ADP-ribose)polymerase
*Prdx1*: Peroxiredoxin-1
ROS: Reactive oxygen species
RNS: Reactive nitrogen species
SNAP: S-Nitroso-N-acetylpenicillamine
*Sod1*: Superoxide dismutase-1
*Srxn1*: Sulfiredoxin 1
tBHQ: * tert*-Butylhydroquinone.
